# The impact of anti-malarial markets on artemisinin resistance: perspectives from Burkina Faso

**DOI:** 10.1186/s12936-023-04705-0

**Published:** 2023-09-13

**Authors:** Rosemonde M. Guissou, Chanaki Amaratunga, Freek de Haan, Fatoumata Tou, Phaik Yeong Cheah, R. Serge Yerbanga, Ellen H. M. Moors, Mehul Dhorda, Paulina Tindana, Wouter P. C. Boon, Arjen M. Dondorp, Jean Bosco Ouédraogo

**Affiliations:** 1 Institut de Recherche en Sciences de la Sante, Centre National de la Recherche Scientifique et Technologique, Ouagadougou, Burkina Faso; 2 Mahidol Oxford Tropical Medicine Research Unit, Faculty of Tropical Medicine, Mahidol University, Bangkok, Thailand; 3 Center for Tropical Medicine and Global Health, Nuffield Department of Medicine, University of Oxford, Oxford, UK; 4 Copernicus Institute of Sustainable Development, Utrecht University, Utrecht, The Netherlands; 5 Institut des Sciences et Techniques, Bobo-Dioulasso, Burkina Faso; 6 School of Public Health, College of Health Sciences, University of Ghana, Accra, Ghana

**Keywords:** Malaria, ACT, Artemisinin resistance, Antimalarial market, Burkina Faso

## Abstract

**Background:**

Widespread artemisinin resistance in Africa could be catastrophic when drawing parallels with the failure of chloroquine in the 1970s and 1980s. This article explores the role of anti-malarial market characteristics in the emergence and spread of arteminisin resistance in African countries, drawing on perspectives from Burkina Faso.

**Methods:**

Data were collected through in-depth interviews and focus group discussions. A representative sample of national policy makers, regulators, public and private sector wholesalers, retailers, clinicians, nurses, and community members were purposively sampled. Additional information was also sought via review of policy publications and grey literature on anti-malarial policies and deployment practices in Burkina Faso.

**Results:**

Thirty seven in-depth interviews and 6 focus group discussions were conducted. The study reveals that the current operational mode of anti-malarial drug markets in Burkina Faso promotes arteminisin resistance emergence and spread. The factors are mainly related to the artemisinin-based combination therapy (ACT) supply chain, to ACT quality, ACT prescription monitoring and to ACT access and misuse by patients.

**Conclusion:**

Study findings highlight the urgent requirement to reform current characteristics of the anti-malarial drug market in order to delay the emergence and spread of artemisinin resistance in Burkina Faso. Four recommendations for public policy emerged during data analysis: (1) Address the suboptimal prescription of anti-malarial drugs, (2) Apply laws that prohibit the sale of anti-malarials without prescription, (3) Restrict the availability of street drugs, (4) Sensitize the population on the value of compliance regarding correct acquisition and intake of anti-malarials. Funding systems for anti-malarial drugs in terms of availability and accessibility must also be stabilized.

## Background

Malaria remains a major cause of mortality and morbidity in African countries including Burkina Faso [1]. Although effective therapies have for long been available to treat malaria, these treatments are threatened by the emergence and the spread of anti-malarial drug resistance. Historically, the approach to address anti-malarial drug resistance is changing the treatment policy to an alternative therapy when treatment failures are observed [2, 3]. Faced with the emergence of resistance to conventional monotherapies for the treatment of uncomplicated *Plasmodium falciparum* malaria, in the early 2000s the World Health Organization (WHO) recommended a switch from conventional monotherapies to artemisinin-based combination therapy (ACT). A first technical consultation in 2001 eventually led to recommendations towards ACT deployment in 2006 [4, 5]. While ACT was gradually implemented in affected regions of Southeast Asia, its introduction was delayed due to financial, political and logistical difficulties in most African countries [3, 6, 7].

Unfortunately, anti-malarial drug resistance is a concern once again, with emerging artemisinin and partner drug resistance. Artemisinin partial resistance was reported in Cambodia in 2009 and has now emerged or spreaded across several countries in Southeast Asia [8–12]. Worrisome reports of artemisinin partial resistance were recently reported in Rwanda and Uganda, although both countries report that ACT still remains efficacious [13, 14]. The consequences of widespread artemisinin resistance in Africa could be catastrophic when drawing parallels with the widespread failure of chloroquine in the 1970s and 1980s. While great efforts have been made over the past decades to reduce the global burden of malaria-related morbidity and mortality, further emergence or spread of artemisinin resistance and subsequent ACT failures will threaten these recent successes [1, 15].

Alternative drug compounds to treat malaria are being developed, but they are not yet available [3, 16, 17]. Therefore, new therapeutic regimens and strategies should make use of currently available drug compounds [18]. In addition to rotating the artemisinin-based combinations and extending the duration of treatment of ACT, the introduction of triple artemisinin-based combination therapy (TACT) and using multiple first line therapies are being explored as strategies making use of current anti-malarial drug compounds, which could delay resistance [18–22].

The link between anti-malarial drug deployment policies and behaviours, and anti-malarial resistance has been widely demonstrated [3, 23–26]. Unregulated utilization of drugs has been identified as the main misuse behaviour that promotes anti-malarial drug resistance. Socio-economic factors and (un)availability of therapies have also been related to resistance along with health system weaknesses and supply chain deficiencies [26, 27]. Anti-malarial market characteristics, including supply chain processes, quality control systems, distribution procedures and storage conditions, have previously been analysed, showing their impact on the quality, price and/or the availability of anti-malarial drugs [6, 28–32].

The link between the anti-malarial market characteristics and the emergence and spread of artemisinin resistance has however been under-investigated, especially in the current context of the emergence of artemisinin resistance in African countries. Studying this link in a high-transmission country like Burkina Faso can enhance understanding of factors– global or country-specific—that could contribute to the emergence and spread of resistance to artemisinin and ACT partner drugs, as well as factors that could protect future therapies.

This study sought to describe anti-malarial drug market structures in Burkina Faso from a systemic perspective, taking into account a comprehensive set of actors, institutions and networks. It explores characteristics of the anti-malarial drug markets in Burkina Faso in terms of policies, distribution, and deployment mechanisms, and their relationship to potential emergence and spread of artemisinin resistance, in order to define better regulation of market-related processes to preserve current and future treatment options.

## Methods

### Study setting

Burkina Faso reported over 10 million uncomplicated *P. falciparum* malaria cases in 2020, and 94.76% of these cases were treated with artemether-lumefantrine (AL) [1, 33]. This reflects the widespread use of AL in the country. Burkina Faso is firmly committed to the fight against malaria through the “Programme National de Lutte contre le Paludisme”, the National Malaria Control Programme (NMCP) created in 1991 and aims to eliminate malaria by 2030. Among its main activities are the development of national guidelines for the management of malaria, surveillance of the disease, and the management of the supply of drugs and products against malaria.

### Supply of anti-malarial drugs in Burkina Faso

The National Agency for Pharmaceutical Regulation “Agence Nationale de Régulation Pharmaceutique” (ANRP) oversees regulating drugs in Burkina Faso via granting a marketing authorization (MA), i.e. a license for any drug to be legally marketed in the country. MA is compulsory for all anti-malarial drugs sold in the country and is granted in accordance with recommendations made by the WHO and the West African Monetary and Economic Union (UEMOA). ANRP, in collaboration with the National Public Health Laboratory (LNSP), monitors the pharmaceutical market, through quality control and pharmacovigilance processes. Similar to other pharmaceutical products, supply of anti-malarial drugs is ensured through two main channels [34, 35]:

- A purchasing center for Essential Generic Medicines (EGM), the CAMEG “Centrale d’Achat Médicaments Essentiels Génériques”, which is a non-profit organization with a public service mission, i.e. providing the majority of the population with quality medicines at lower cost. CAMEG is in charge of supplying pharmaceutical depots and pharmacies of all public health structures, and also interested private pharmacies, with EGM and medical consumables. It orders, stores and manages all the drugs (generics drugs) purchased for government routine activities or for implementation programmes (such as malaria, HIV, tuberculosis). All the technical and financial partners (TFPs), including the Global Fund to fight Aids, Tuberculosis and Malaria (GFATM), fundingsgo through CAMEG to supply public health centers with EGM and other essential commodities such as Rapid Diagnostic Test (RDTs) for malaria. Through its 10 commercial agencies, CAMEG supplies approximately 70 dispatchers’ deposits and 1698 public pharmaceutical depots.
- Private wholesale distributors are both in competition and complementarity to the activities of CAMEG. Four of them are prominent: Société de distribution pharmaceutique du Burkina Faso (DPBF), Laborex, Tedis pharma and Ubiphram. Their headquarters are situated in Ouagadougou, with branches located in Bobo-Dioulasso. These private wholesalers are responsible for supplying private pharmacies with brand-name drugs but also with some generic drugs under license, which are copies of brand names and called "branded generics". They supply approximately 274 private pharmacies and 527 pharmaceutical depots.

### Malaria care delivery in Burkina Faso

National malaria management guidelines developed by the NMCP are mainly based on WHO recommendations [36–38]. Disease management starts with a biological diagnostic test (rapid diagnostic test or microscopy) followed by anti-malarial treatment: oral treatment for uncomplicated malaria and an intravenous route for severe malaria. Therefore, anti-malarial drug consumption should only occur after a consultation and/or a diagnostic test by a health worker. Since 2005, following WHO recommendations, the country adopted ACT for the management of uncomplicated malaria.

Artemether-lumefantrine (AL), dihydroartemisinine-piperaquine (DHA-PPQ) and artesunate-pyronaridine (AP) are the artemisinin-based combinations recommended by the NMCP and Ministry of Health [39]. Anti-malarial drugs with an MA are not necessarily in the NMCP’s guidelines. The public sector solely uses anti-malarial drugs recommended by the NMCP. This sector, which is the main health care provider in Burkina Faso, is organized under a pyramidal form at three levels providing primary, secondary and tertiary health care. Primary health care are deliver at local levels mainly by Health and Social Promotion Centres and Medical Centres with surgical services. Regional and University/National hospitals deliver respectively secondary and tertiary health care. Burkina Faso also has a private health sector, which is mostly concentrated in urban areas, with medical clinics and nursing offices. This sector also includes denominational structures which are not-for-profit structures and therefore offer services at social prices.

### Access to anti-malarial drugs by patients in Burkina Faso

According to regulations, access to anti-malarial drugs is conditional on the presentation of a prescription. However, in practice, access to anti-malarial drugs is often independent of a medical prescription and/or a diagnostic test [34]. Patients’ access to medicines, including anti-malarials, in Burkina Faso’s health system is achieved through pharmaceutical depots or pharmacies, which are found both in the public and the private sectors.

In the public sector (and often for denominational health structures), each structure of the three levels has a pharmaceutical depot or a pharmacy in which generic anti-malarial drugs are available for purchase. Artemether-lumefantrine (AL), is the main artemisinin-based combination used in the public health sector for the management of all ages’ uncomplicated malaria [1, 38]. AL is provided for free to pregnant women and children under 5 years of age. For the rest of the population, it is available at a subsidized price in public health structure drug shops. This policy, financed by the government and its TFPs, make generic versions of artemisinin-based combinations better accessible in comparison with specialties and their copies.

Private, for-profit outlets are also important treatment sources for malaria in Burkina Faso [34, 35]. There, patients have access to all anti-malarials that have an MA, but are not necessarily recommended through NMCP guidelines. Private pharmaceutical depots are generally located in rural or semi-rural areas and are owned by pharmacies that are established in urban areas. Consumer demand, influenced by drug prices and patient preferences, is their principal consideration when selecting products to stock [34].

Traditional therapeutics that provide treatment for malaria through administration of medicinal plants are also available. Burkina Faso has taken efforts to regulate this ancestral practice by setting up a Directorate of Traditional Medicine and Pharmacopoeia in 2022, which aims to regulate the promotion and enhancement of traditional medicine. This support is manifested by the sale of some of its traditional pharmacopoeia products in pharmacies.

Pharmaceutical products in Burkina Faso are also sold on the stalls of open-air markets as "street drugs"; they include both counterfeit drugs and drugs from the normal circuit [40, 41]. In the latter situation, the storage conditions are often considered suboptimal, which causes the substance to lose its attributes, therefore its stability and its effectiveness. Unlike in official pharmacies and depots, they are available for retail sale (as individual tablets or full doses) and their prices are negotiable. They are also more easily accessible because the sellers base themselves in the customer's living environment: in streets or in market/shops stalls. Lower cost, easy access and availability are the advantages of these products, and despite recognizing the dangers imposed by them, consumers continue to use them [40, 42].

### Study design and population

This study was conducted under the auspices of the ‘Development of Triple Artemisinin-based Combination Therapies (DeTACT)’ project. A mixed methods approach using in-depth interviews (IDIs) and focus group discussions (FGDs) in combination with a literature review was used for this study [43]. A representative sample of national policy makers and regulators, public and private sector wholesalers and retailers, clinicians, nurses, and community members were purposively sampled for semi-structured IDIs and FGDs and they were asked to answer questions related to the market positioning and ethical concerns regarding potential deployment of triple ACT (Table 1**)**. The details of the methodology have been previously described [43, 44]. Data collected additionally revealed many aspects of anti-malarial drug market dynamics in Burkina Faso and were probed for this study. Added information was obtained from a literature review of peer-reviewed manuscripts, policy publications and grey literature on anti-malarial policies and deployment practices in Burkina Faso.

**Table 1:** Respondents in Burkina Faso

Respondent categories		N		Interview type
		Ouaga	Bobo	
Policy maker	National Agency for Pharmaceutical Regulation “Agence Nationale de la Regulation Pharmaceutique” (ANRP)	**2**	**–**	Interview
	National Malaria Control Program (PNLP)	**3**	**–**	Interview
	Regional health authority	**2**	**1**	Interview
	Malaria researchers	**2**	**1**	Interview
Drug wholesaler and retailer	Public sector wholesalers	**3**	**–**	Interview
	Private sector wholesalers	**–**	**6**	Interview
	Public sector retailers	**–**	**2**	Interview
	Private sector retailers	**1**	**2**	Interview
Drug end user	Public sector health care providers	**1**	**3**	Interview
	Community based health workers	**–**	**1**	FGD
	Private sector health care providers	**5**	**3**	Interview
	Parents/Caregivers	**–**	**5**	FGD

Bold values are the number or individual interviews and/or focus discussions groups conducted

### Data collection

Five semi-structured guides were developed for the different targeted groups [43]. Data collection was carried out by two trained and experienced social scientists, under the supervision of a sociologist (FT) and a health economist (RMG). FGDs were conducted with groups of 8–10 participants. Collection sites were Ouagadougou (the capital), Bobo-Dioulasso (the second largest city in the country) and the village of Santidougou, a rural village situated 17 km away from Bobo-Dioulasso. Data collection was conducted from March to April 2020 in Bobo-Dioulasso, and in June 2020 in Ouagadougou.

### Data processing and analysis

Interviews were transcribed under the supervision of the team's sociologist who subsequently analyzed them. Using NVivo 10 software, each transcript was coded according to pre-identified themes. Some themes were identified during the study design (inductive) but some themes also emerged during the analysis (deductive). In the study design, the main themes were malaria control strategies in the country and country transition from monotherapies to ACT (described previously in part in the methods section because they pertain to the study setting), and anti-malarial drugs market operating mode (process for obtaining marketing and importation authorizations, distribution channels, utilization). Respondents’ opinions on the threat of artemisinin resistance and strategies to deal with it emerged as a theme during the analysis.

## Results

Data from 37 semi-structured in-depth interviews (IDIs) and 6 focus group discussions (FGDs) were analyzed to explore anti-malarial market characteristics and their potential effect on emergence or spread of artemisinin resistance in Burkina Faso. Policies and processes associated with ACT supply process including quality control, ACT prescription and use practices, and respondents’ views on their implications to potential artemisinin resistance in Burkina Faso were analysed and discussed.

### Anti-malarial drugs supply process in Burkina Faso

As described previously, there are two main supply channels for authorized anti-malarial medicines, predominately artemisinin-based combinations, in the country: via CAMEG for generics with government as main client, and via private wholesale distributors for brand-names and branded generics. Disruptions in ACT supply chains, weaknesses with regards to the quality control process of ACT, and reduced availability of generic ACT were identified as supply-side characteristics that can influence emergence and spread of artemisinin/anti-malarial drug resistance in Burkina Faso.

### Supply chain of ACT

Disruptions in supply chains are observed in both generic and brand-named artemisinin-based combinations. For the generics, private wholesalers assert that the state has given the monopoly of the supply and distribution to CAMEG, and this leads to frequent disruptions in the supply because of it reduced capacities. [«*This is really one of the problems we are currently experiencing. CAMEG monopolizes generics [*supplying*] when it is unable [*for it*] to respond to the market [*demand*]*» Supplier 5, Private sector wholesaler]. A manager of the CAMEG refuted this idea and affirmed that private wholesalers have the right and can import generic ACTs, but they did not do this because they are motivated by profit margins. [«*[…]* …. *So, I would say it's not a question of monopoly but a question of choice*.» Supplier 2, Public sector wholesaler]. Another CAMEG manager and a respondent from the NMCP explains, indeed, that a large part of generic ACTs supply disruptions is due to the late reimbursements of the government to health structures as part of its free policies, which impacts the resupplies. [« *We say free, but someone pays. […] if that person doesn't pay on time, […] it goes without saying that we are now accumulating what we call receivables and receivables are reflected in debts vis-à-vis suppliers since we also buy on credit. And when the debts are high it decreases our negotiation capacities to receive the products*.» Supplier 1, Public sector wholesaler].

Shortages observed in the supply of brand-name drugs and their copies, are explained by private wholesalers by administrative burden in the procurement. [« *[…], it's mainly at the customs level: customs negotiations […]. Sometimes it's not so much the product that you don't find the product is there, but it is in a container that is sleeping. [….] The problem is the procedure, it's not so much the amounts. […] We drag out disruptions not because the product is lacking at the international level but because there is bureaucracy»* Supplier 6, Private sector wholesaler].

Drug availability is an essential element in disease management. Disruptions in availability provokes inadequate and incomplete treatment and misuse behaviours. It also enhance street drugs consumption and self-medication behaviours among the population. All of those behaviours subsequently promote anti-malarial drug resistance.

### Quality control process of ACT

According to respondents, drug quality control systems are successfully integrated into the CAMEG supply process, but for the private wholesalers, the situation is different. Respondents indicated that there are too many ACT brand-names and their copies granted with a marketing authorization (MA). [*« It's true, we're talking about competition, but I want the regulations to limit the number of brand-names to 25 or 30»* Supplier 5, Private sector wholesaler]. Respondents from regulatory authorities recognized this issue [*« […] otherwise it is a real concern. This has led us to try to [actually] limit registrations of the same INNs [*International Nonproprietary Name*]»* Policy maker 1, ANRP]. Given the limited quality control capacities of the ANRP, this has an influence on the (perceived or real) quality of drugs found in pharmacies. [*« What I have as complaints is the multiplicity of forms existing on the ground, […] even if these are the same combinations the sources are multiple and the brands also, so that the populations wonder if their quality and effectiveness is no different. [….]»* Supplier 1, Public sector wholesaler]. *[« […] the national laboratory has to carry out checks/samples before selling but I do not think this is done everywhere. [……]. I know that there are checks that are done and the results come to find that the selling is finished»* Supplier 5, Private sector wholesaler].

Street drug artemisinin-based combinations that are easily accessible, despite prohibiting laws, exacerbates this problem [*«…. I know what I am talking about, I assure you that there are counterfeit antimalarials on the Bobo market: full, overdosed and underdosed»* Supplier 5, Private sector wholesaler]. A pharmacist in a private wholesaler company explained that the sales strategy of this street market attracts users: prices can be negotiated, and drugs can be obtained in retail facilities, which is not the case in conventional pharmacies.

Besides patient health and safety threats, non-quality assured ACT also affect artemisinin efficacy, and sub-optimal dosing provides a favourable environment for the emergence and spread of resistance.

### Number of generic ACT used in public health sector

Only one main artemisinin-based generic combination, AL, is used in the public sector in Burkina Faso, as first line treatment for the management of uncomplicated malaria. A respondent explains that there are other combinations in national guidelines that work well but they are too expensive compared to AL. Extensive use of drugs is a factor favouring the emergence or the spread of resistance and decision-makers are aware of the threat: [*« From the moment that we use the same drugs for mass campaigns, SMC and such, we should have in mind the history of resistance…*.» Policy maker 3, NMCP].

Due to wide spread use of a single ACT, selection pressure on AL is high and can accelerate the emergence and spread of artemisinin resistance in the country.

### Anti-malarial prescription and use practice in Burkina Faso

The malaria treatment guideline is officially defined at national levels for both the public and the private health sector. Compliance by health workers and by patients are equally important in ensuring good anti-malarial prescription and use practice.

### Accessibility to ACTs, self-medication, and correct use by patient

In legislation, availability of anti-malarial drugs in pharmaceutical depots or pharmacies is conditional to presenting a prescription. In practice, however, accessibility is void of this condition. This leads to a strong tendency to self-medicate, which was denounced by many respondents. For them, self-medication is often associated with treatment discontinuity when the patient feels better, when the patient feels that the product is not working or, because of side effects.

A pediatrician observed: [*« … we are the ones who make parents aware of avoiding self-medication because that is what leads to resistance. They go to the pharmacy, they get served, they start the product, it doesn't work, they stop. [….] the product must be given time to act.»* User 6, Private sector health care provider].

However, another respondent nuanced the situation, stating that self-medication is not the main problem given the current state of the health system, the country should rather sensitize the population on good compliance. [*« If all malaria patients at one time had to go to the prescriber before taking a product, the health service will be crowded. So, at the stage where we are, it's only normal for minor suspicions so that people can treat themselves, whether with medicinal plants or with modern products»* Supplier 1, Public sector wholesaler].

A pharmacist insisted on the need to sensitize patients on correct treatment administration: [*«For me it is necessary to work so that people respect the dosage, because the resistance comes from the fact that we do not respect the times of intake. […], it takes 8 h between the first 2 takes. After the 8 h […], for sure you will distort your treatment and come and say that it is the drug that is not effective and little by little the parasites will get used to the molecule and develop resistance.…»* Supplier 11, Private pharmacist].

Self-medication increases the risk of inadequate treatment, i.e. non-compliance with correct dosage and/or regimen, all of which are recognized as factors that accelerate drug resistance. Nevertheless, given the large burden of malaria, instead of not providing easy access to anti-malarials, it could be important to raise awareness of the short-term effects (poor efficacy for current illness) and long-term effects (anti-malarial drug resistance) of poor compliance during self-medication.

### Health worker’s compliance with national guidelines

Lack of diagnosis and inappropriate treatment prescription are behaviours of health workers commonly observed in Burkina Faso. A researcher stated that the overconfidence of health workers based on their experience leads them to prescribe anti-malarials without diagnostic tests, despite the national guidelines prescribing them. An official at the regional level was indignant: [*«…at the level of* [public] *health facilities, health care provision is criticisable. I have just returned from a supervision, in certain situations the health workers themselves systematically prescribe in case of fever* [without any diagnostic test]*»* Policy maker 8, Regional Health Authority].

Both public and private health worker respondents in the current study claimed to follow national recommendations for malaria management. [*« The same protocols are applied [*in public as in private health sector*] whether for severe or uncomplicated malaria»* User 4, Private health care provider]. When asked if there is a difference in anti-malarial prescription behaviour between the two sectors, they responded affirmatively although with different points: public sector practitioners believe that there are indeed shortcomings at the level of the private sector on the diagnosis which can be explained by their lack of access to rapid diagnostic tests. [*«…in the public, we have access to the RDTs that we use before the anti-malaria prescription, but in the private sector, especially in private practices nursing which we know, most of the time they do not have access to the RDTs. They prescribe according to the symptoms described by the patient.»* User 11, Public health care provider]. For private sector practitioners, prescription behaviour differences between the two sectors lies mainly in the type of drugs prescribed: the public sector favours generic ACT, while the private sector prescribes brand-name drugs or branded generics. [*«…I think it's the same efficiency, it's a question of presentation and also a question of prejudice in relation to patients. It is the patients who guide us in our prescriptions because when you prescribe generics, people have a fixed idea. [….] Imagine if someone makes a consultation of 5000 FCFA and in return you give him a prescription of 200 FCFA. It's weird so that's mainly why we tend to prescribe brand-name in private sector. Otherwise personally, I do not think there is a difference between generic and brand-name drugs.»* User 4, Private health care provider]. They did not address the diagnosis aspect, except one of them, who justified patients’ recourse to private sector by their search for a good diagnosis: that is provided by microscopic examination of a blood film, even though they do not have RDTs.

Several respondents justified the inappropriate treatment/combination prescription as being the result of strong patient preferences. A public sector pediatrician explained that some patients insist on intravenous administration of their medicinal products for treating uncomplicated malaria, arguing that this would promote rapid healing. When they face a refusal in a public health facility, they go to the private sector where they are provided the required service. [*«* ….*They [*Patients*] are in too much of a hurry. There is no miracle drug. It takes time for it to go*.*»* User 6, Private sector health care provider]. To preserve their customers, practitioners often accede to these user requests, particularly in the private health sector. This sector is more prone to non-compliance, which a respondent explained by stressing the almost total absence of quality control within the private sector. In the public sector, regional directorates, sometimes with NMCP collaboration, has the possibility of identifying poor quality diagnostic/prescription behaviours during supervision visits. An official confessed: [*« For private health care center, especially large clinics, we can't manage to supervise at their level; we can't access to ensure, for example, that the diagnosis has really been confirmed before carrying out the treatment, that the [right] molecules have been proposed and respect…»* Policy maker 3, NMCP].

A private pharmacist also insisted on the non-conformity of several anti-malarial prescriptions for which she had to correct the dosage: [*«When you receive a prescription where they put* [non conform dosage]*, it is the duty of the pharmacist to rectify this…»* Suppliers 11, Private pharmacist].

Inappropriate health care management practices by health system actors, with overuse and misuse of drugs can accelerate the emergence and spread of artemisinin resistance.

### Artemisinin resistance in Burkina Faso

Despite the absence of scientifically established results on the presence of artemisinin resistance in Burkina Faso, respondents expressed their views on the subject. For some, it has already been clearly established in the light of observations of therapeutic failures in the field, for others the causes of these failures are to be sought elsewhere.

### The case for prevalence of artemisinin resistance in Burkina Faso

Based on their personal observations, complaints from clients, patients or relatives, some of the respondents believe that therapeutic failures with ACT are prevalent, reflecting the emergence of artemisinin resistance. One of them questioned: [*« Why before that gave with the same treatments and today, it is necessary to lengthen the treatment or even it is necessary to change treatments? We do a treatment, it does not work, we change molecules…»* Policy maker 2, ANRP]. A wholesaler who is also pharmacist explained: [*« […] in pharmacies there are several customers who come and ask for another malaria treatment. I tell them “dear friend, 4 days ago you were here for an antimalarial.” He says he feels he is not healed. Isn't there anything else? So the resistance, I don't know if it's psychological, but it already exists here. It may not be strong but it exists»* Supplier 6, Private sector wholesaler]. For them, it is timely to introduce another combination.

### The case against prevalence of artemisinin resistance in Burkina Faso

Other respondents were more cautious and link these observations of presumed therapeutic failure to the misuse of ACT. According to them, it would be necessary above all to invest in sensitization of the population on self-medication as well as in sanitation, the promotion of good protection measures against mosquitoes (e.g. mosquito nets) [*« Frankly, tell them* [decision makers] *to focus on environmental sanitation is the only thing that can save us from malaria. Medicines are useless, primary prevention is what we must focus on…let's make sure there are no more mosquitoes…»* User 6, Private sector health care provider]. A researcher stated: [*«Never change a winning team! If ACTs are still effective in Burkina Faso, I don't know why the therapeutic relentlessness is necessary. We cannot leave drugs that are good to jump to other combinations»* Policy maker 9, Malaria researcher].

## Discussion

Anti-malarial drug market characteristics in Burkina Faso were explored to understand how they might accelerate or prevent the emergence and spread of artemisinin resistance in the country in the context of an imminent threat of artemisinin resistance in African countries. The study shows that the current operational mode of anti-malarial drug markets promotes resistance. This requires rethinking some aspects of current anti-malarial pharmaceutical policies and health system characteristics. These aspects are mainly related to (a) ACT supply chain disruptions, (b) weaknesses in quality and prescription monitoring, (c) uncontrolled access to anti-malarials and misuse by patients.

- ACT supply chain disruptions

A robust structure of the anti-malarial market supply in Burkina Faso prevails, with two mains official channels: a central purchasing with a public mission (CAMEG) in charge of generic ACT procurement mainly for public health structures, and approved wholesalers who supply private markets with brand-name ACT and branded generics.

Centralization of procurement has proven to reduce drug stock-outs and increase drug availability for populations [45]. Large quantities ordered at central locations offers economies of scale and leads to affordable acquisition and sale prices. However, centralization has its limitations, given the problems in ACT availability, highlighted by respondents especially in the public health sector. Supply disruptions seem to be a trend in low- and middle-income countries (LMICs). An evaluation of availability rates of generic drugs for acute conditions in basic health structures of 40 LMICs revealed a mean availability of 53.5% in the public sector and 66.2% in the private sector [46]. In Brazil, this rate was estimated at 52.9% in 2017 [47]. Weaknesses in purchasing and/or supply management processes and inadequate funding systems generally explain disruptions in the public health sector [45, 48, 49]. Similar explanations emerge from our results and are confirmed by national data that show a dramatic increase in the percentage of pharmaceutical depots that have experienced a shortage of generic ACT: 9—23% in 2010–2015, increased to 72% in 2016 and 85.5% in 2020 [33, 50, 51]. The numbers from 2016 correspond to the beginning of implementation of the policy of free healthcare for children under 5 and pregnant women, including malaria care. The deficits in ACT at the beginning of implementation of this policy can be explained by the period of adaptation of capacities, i.e. a steep increase in demand due to care becoming free. The persistence of this deficit in the following years confirms dysfunctions at the level of logistics and financing systems. CAMEG supply chain management capacities must of course be strengthened. However, the most urgent need according to the present study, is to secure funding sources for generic ACT, especially for no-charge policies. For the private sector, strengthening customs service’s knowledge on pharmaceutical products will contribute to improving administrative processing times and avoid disruptions observed in ACT brand-names and their copies.

ACT supply disruptions have an influence at several levels on drug-use behaviours. At the health worker level, if recommended combinations are not available, they will prescribe whichever anti-malarials are available, regardless of what national healthcare guidelines recommend. Replacement of continuous-use medicines by unregulated drugs may compromise the control of the disease and/or the adherence to therapy, affecting the treatment effectiveness [47]. At the patient level, disruptions can impact adherence to treatment or lead to renunciation. This can also enhance the use of street drugs, which are cheaper than those obtained at private pharmacies [30]. All these misuse behaviours, generated by disruption of ACT supply can accelerate the emergence and spread of artemisinin resistance.

b) Weaknesses in quality and prescription monitoring.

To ensure drug safety, it is important that drug quality and proper prescription are safeguarded at the national level. In Burkina Faso, shortcomings in the quality control process have been observed regarding the quality of drugs and their prescription practice, particularly in the private sector which provides a significant part of the country's supply of medicines. Additionally, there is pressure exerted on the main ACT used at the national level, due to lack of other affordable ACTs and the policy of a single first-line treatment.

### Weaknesses in quality control of ACT imported by private sector wholesalers

According to the WHO, 1 out of 10 medical products was reportedly counterfeit or substandard in LMICs and most of them were anti-malarials or antibiotics [52]. A study conducted in eight African countries showed that non-quality assured ACT accounted for 8–40% of the market share in the private sector drug stores, and this trend was much weaker in the public sector [30]. Burkina Faso government’s weak ability to control the quality of imported ACT is highlighted by study results and is all the more worrying as their quality is often questionable, especially in the case of street drugs. Tinto and Rouamba (2020) explained that many drugs of questionable quality or counterfeit drugs are circulating on the Burkinabe market and the country does not have the technical platform to address this critical health problem [53].

Availability and use of poor quality anti-malarials have historically been linked to treatment failure and in some cases, have coincided with the emergence or spread of resistance [54]. There have been alarms of anti-malarial resistance that were in reality due to poor-quality anti-malarials and not due to parasite resistance [16]. Efficacious anti-malarials are crucial for malaria control and it is essential to monitor their efficacy in order to inform treatment policies and detect, as early as possible, emerging drug resistance [3]. Drug quality control and pharmacovigilance systems for private sector are important issues to address to delay the emergence or the spread of artemisinin resistance in Burkina Faso. ANRP appears to be moving towards reducing the number of ACT imported by wholesalers in order to allow concentrated and more effective controls. Indeed, the multiplicity of supply sources for private wholesalers, denounced even by Burkinabe private pharmacies owners [34], makes control more difficult. The same observation was made in several other African countries with a listing of 92 different suppliers in Nigeria [30].

While waiting to be able to set up an effective quality control system, ANRP could rely on the WHO prequalification programme which ensures the quality of drugs in the public sector. This programme certifies non-toxicity and efficacy of drugs and is mandatory for any transaction carried out with international funding, as is the case for a large part of generics ACT purchase in Burkina Faso [6, 55]. With the centralization of purchases by CAMEG, a sufficient level of quality control of generic medicines is ensured. If some brand-names and their copies are in this prequalification list, they may be prioritized by the government as the quality label is already assured. Restricting the import of brand-names and their copies will require an effective campaign to sensitize private sector vendors, as it is likely to influence their profit margin on these products [29]. Prescribers and patients should also be informed about the risks of the emergence of artemisinin resistance and its consequences and the subsequent importance of adhering to recommended products, insofar as their choice is one of the main factors that guides ACT purchases by private wholesalers [29].

Anti-malarial market actors in Burkina Faso recommended strengthening the fight against street drugs by the government, even though literature review suggested that artemisinin-based combinations were not present among street drugs in the country [41, 56]. Nevertheless, the timeline of previous reports (2006–08), the existence of counterfeit ACT in neighbouring countries [30] and the high rate of access of this market by the population [40, 41] compels us to conclude that there is a strong probability that ACT of poor quality are available in the streets of the country. Financial and geographical accessibility as well as the possibility of unit purchase and price negotiation are the main explanatory reasons for the recourse to street drugs according to our study and this is confirmed by other studies in the African context [26, 40, 42, 57]. Increased and—above all—continuous repression of supply and sales circuits (including markets, street vendors) would certainly reduce availability of street drugs. Numerous repression operations have been carried out, yet a lack of continuous follow-up seems to suggest an absence of political will. At the same time, the Burkinabe government tried to strengthen the alternative to street drugs more appealing by providing stable and secure funding system in the form of a policy for better access to quality-assured ACT. This has been shown in 8 countries of sub-Saharan Africa, demonstrating increased accessibility to ACT especially in hard-to-reach areas [58].

### Weaknesses in quality control of ACT prescription

Apart from the quality of the drug, its proper use is necessary for an effective and efficient result. Even if health workers, being from public or private health sectors, claim to follow national recommendations, respondents in this study raise questions about the quality of malaria management and anti-malarial prescriptions. They highlighted a lack of diagnosis before treatment administration and prescription of inappropriate treatment/combination as well as the lack in the control of practice, particularly in private sector. The study results are consistent with several studies that have reported sub-optimal health worker compliance with ‘test and treat’ malaria guidelines in endemic sub-Saharan African countries including Burkina Faso [26, 59–63]. Malaria treatment is still largely presumptive despite the recognition of the usefulness of diagnostics tests and their increasing availability, particularly in public health sectors [58–61]. The findings also show an absence of health care practitioners’ quality control by Burkinabe government’s, despite it being in the texts, especially in the private sector. Even training on compliance to national guidelines is scarce, the government usually contenting itself with sending them paper versions of national directives, while this sector is the most indexed in the literature for is inability to diagnose and treat non-malarial fevers, and an innate motive to over-prescribe malaria treatment [64].

Weak financial capacity is put forward by regulators as one of the reason for the lack of quality control of health care practitioners with regards to malaria diagnosis and treatment. However, given the preponderant role of the private sector in the provision of care, it is more than necessary to undertake effective and efficient training and control activities in order to ensure that private sector acts in line with state priorities. RDTs should also be available at their level to enhance their use and improve malaria case management.

The quality of prescription is partially determined by the quality of prescribers. The Burkinabe health system delegates prescribing medication to nurses, community-based health workers and often less qualified health workers in peripheral health centers. This practice aims to facilitate access to care for populations, especially those in rural areas due to lack of doctors. Inappropriate prescriptions observed in the management of malaria is partially explained by this delegation of prescription, without providing adequate training or lack of retrainning [34, 53, 65]. The country is currently in the trend of increasing the availability of doctors at the level of basic public health structures, and this will undoubtedly contribute to improve the quality of prescriptions.

Prescribers’ skill level and experience, together with training and supervision are a necessity to ensure implementation and maintenance of good prescription practices [66, 67]. Inappropriate malaria health care management practices by health system actors result in overuse and misuse of ACT [3, 23, 24].

### Pressure due to limited ACTs used in the country

A study in Burkina Faso of private pharmacies showed that AL was the main anti-malarial dispensed, both with and without a medical prescription, over several years [34]. Our results also highlighted the strong use of generic versions of this combination in the public health sector. The intense utilization of this ACT places selective pressure on artemisinin and the partner drug [20, 24, 68]. Development of new combinations such as triple artemisinin-based combination therapies (TACTs) are being explored as a possible alternative [43, 44]. Multiple first-line therapies (MFTs) are also proposed to mitigate the effects of this pressure and thus delay the emergence and spread of resistance to artemisinin [20, 68, 69]. Beyond questions of efficacy, cost and cost-effectiveness must also be considered; our study confirms that it is this factor that determines the choice of AL combination among the other combinations recommended by the WHO for uncomplicated malaria management.

The Burkina Faso government also encourages and supports the development of traditional medicine, which is widely used by the populations and even medicine practitioners [70–72]. The country’s weak pharmacovigilance capacities explains the low number of traditional drugs available in pharmacies given the large number of medicinal plants used in the management of malaria [70, 73–75]. Continued support for pharmacovigilance activities is required to have safe products that will diversify the medicines offered and reduce the selection pressure on modern anti-malarial combinations.

c) Uncontrolled access to anti-malarials and misuse by patients.

Despite clear regulations, anti-malarials are accessible without prescription at all drug sales outlets in Burkina Faso, similar to many malaria-endemic countries [29]. Free access to anti-malarials leads to high rates of self-medication according to the study respondents. This situation is common in endemic countries since the population has a relatively good knowledge of symptoms, causes, mode of transmission, etc. related to malaria [76, 77]. A survey conducted in Burkina Faso urban private pharmacies among 1,467 people who came to buy an anti-malarial, revealed that 2/3 had come directly, without a prescription [34]. A high rate of self-medication has also been observed in the Democratic Republic of Congo with 96 to 98% of respondents in studies admitting to self-medicate with anti-malarials [78, 79]. In contrast, a study in Ghana reports a lower proportion (16.8%) of non-prescribed anti-malarial use, and explains it by the existence of National Health Insurance Scheme [77]. The direct recourse to treatment is generally not carried out following a diagnostic test. Therefore, there is a risk of treating the wrong disease since diseases highly prevalent in countries with high malaria endemicity, such as dengue fever or typhoid fever, have symptoms very similar to malaria [76]. In addition to inappropriate malaria treatment‐seeking behaviours, this open access also enhances anti-malarial misuse behaviours: use of non-recommended molecules, failure to respect intake intervals, failure to complete the treatment. Several studies have highlighted these types of behaviours in anti-malarial use and have shown their link with the emergence or the spread of resistance: anti-malarial drug resistance has been accelerated by the way drugs are used (or misused) and by the social and economic conditions in which they are used [3, 25, 53, 77, 80, 81]. This is similar to the case of antibiotics misuse, which is widespread with consequences of resistance emergence or spread [82, 83].

### Could requiring a prescription for access to ACT curb these misuse behaviours?

Many study respondents think it could and they suggest it should be regulated. It is also the conclusion of studies on pervasive drug misuses in the same context [79, 82]. However, one interview respondent stated that open access and self-medication is not the problem given country health system organization and access difficulties: the prevalence of malaria in Burkina Faso is such that if all patients visit the health centers, the already insufficient health centers would be even more overwhelmed. This difficult physical access to health services due to their insufficient geographical coverage is a reality shared by LMIC populations. In addition, the aspect of increased financial burden must be considered. If ideal health services were available, this would imply the payment for consultation, diagnostic examination (in the event of recourse to the private sector), and treatment. Burkina Faso, similar to many other LMICs, has a low rate of coverage by health insurance and, therefore, the majority of the populations would bear this burden from their families budgets [82, 84]. Poverty affects integral aspects of malaria treatment-seeking behaviours, including adherence to treatment [23].

Given the structural barriers to anti-malarial access and to proper use behaviour, which cannot be resolved immediately, and with the threat of resistance to artemisinin, Burkina Faso, like other LMICs, should consider strong sensitization campaigns on good compliance in malaria treatment administration and use. Awareness should also focus on compliance with national recommendations for care with the use of quality-assured molecules in order to fight against the use of street drugs. Community members in Burkina Faso generally have a good knowledge of malaria. However, their knowledge of anti-malarial drug resistance is poor [81]. Increasing their knowledge regarding the consequences of the misuse of ACT, on their current illness and at a more holistic level, on the emergence or spread of resistance and their implications such as the cost of a change in treatment at the national level, could promote safe behaviour. Official drug sellers should also be made aware of those aspects with the addition of RDTs being made available to them [34, 59]. The mean cost of RDTs in private Burkina Faso pharmacies was estimated at 1,523 FCFA (2.412 USD), a higher cost than generic ACT in these stores [34]. Supplying RDTs free for the populations would be ideal. Indeed, the combined healthcare costs of both the RDTs and ACT represents a significant barrier to patients in the community, especially in rural areas [85].

### Study limitations

The main limitation of this study is absence of deep investigation into the role of ACT costs and price on anti-malarial market dynamics. Indeed this variable was found important in securing the generic ACT supply chain, on the choice of artemisinin-based combinations for national policies and on anti-malarials use behaviours by community members. These community members are end users of ACT and their support is essential for the successful implementation of an efficient pharmaceutical policy. Given the widespread poverty in Burkina Faso, similar to most African sub-Saharan countries, knowledge of price impact on their choices is important. A system that relies on community members' ability to pay for appropriate treatments risks a repeat of events associated with chloroquine resistance, where an effective and cheap anti-malarial drug was rendered useless partly due to under-treatment [64]. Also, the impact of storage conditions on the quality of ACT, highlighted by the literature, was not investigated in this study. The cumbersomeness of customs administrative procedures on brand supply shortages that emerged in the results, was also not taken into account in the identification of the main respondents.

## Conclusion

This study presents a compilation of important information regarding anti-malarial market characteristics and their possible influence on the emergence and spread of artemisinin resistance in Burkina Faso. Many characteristics of the current anti-malarial market in Burkina Faso are accelerating factors to this effect. These include the ongoing disruptions in ACT supply chain at both public and private wholesalers’ level, weaknesses in quality control systems for ACT, selection pressure on ACT that are used for malaria management, uncontrolled access to ACT by patients, and misuse of ACT by both prescribers and community members. Most of these dysfunctions are structural barriers that cannot be resolved in the short to medium term even though the threat of artemisinin resistance looms. Malaria control in African malaria endemic countries is mainly based on treatment interventions. Therefore, it is necessary to have an effective and efficient drug policy design that takes into account local constraints. The malaria research community is working on the modification of existing treatments, as well as the discovery and development of new drugs to counter resistance to current drugs. While waiting for these innovations, solutions that can be quickly implemented must be considered. The majority of study respondents recognized an increase in therapeutic failures. They are aware of the risk of the emergence and spread of artemisinin resistance in Burkina Faso and associate this with factors related to anti-malarial markets and subsequent (mis) use of anti-malarials. To address this threat, four recommendations for public policy emerged during data analysis: (1) Address the suboptimal prescription of anti-malarial drugs, (2) Apply laws that prohibit the sale of anti-malarials without prescription, (3) Restrict the availability of street drugs, (4) Sensitize the population on the value of compliance regarding correct acquisition and intake of anti-malarials. Funding systems for malaria treatment facilitate the availability and accessibility of appropriate therapies for the maximum number of people and must, therefore, be stabilized. The dramatic consequences, both in terms of economic and public health outcomes, of the emergence and the spread of chloroquine resistance must not be repeated with ACT.

## Data Availability

There are ethical and legal restrictions to sharing our data publicly but they are available upon request from MORU Data Access Committee (https://www.tropmedres.ac/units/moru-bangkok/bioethics-engagement/data-sharing). Most interviews are directly traceable to individual identities and therefore we cannot share the interview data without releasing the identities of the respondents. In each interview, respondents introduced themselves and spoke about their direct (working) environment. Moreover, the topics discussed and responses to questions, could be directly linked to their job positions and affiliation, especially with higher level policy and regulatory officials. We guaranteed full anonymity to the respondents prior to data collection and therefore sharing the dataset without restrictions would be unethical.

## Abbreviations

ACT: Artemisinin-based Combination Therapy
AL: Artemether-Lumefantrine
ANRP: National Agency for Pharmaceutical Regulation / Agence Nationale de Régulation Pharmaceutique
ASAQ: Artesunate-amodiaquine
CAMEG: Centrale d’Achat Médicaments Essentiels Génériques
DeTACT: Development of Triple Artemisinin-based Combination Therapies
EGM: Essential Generic Medicines
FGDs: Focus Group Discussions
IDIs: In-depth Interviews
LMICs: Low- and Middle-Income Countries
LNSP: National Public Health Laboratory / Laboratoire National de Santé Publique
MA: Marketing Authorization
NMCP: National Malaria Control Programme / Programme National de Lutte contre le Paludisme
RDTs: Rapid Diagnostics Tests
SMC: Seasonal Malaria Chemoprevention
TACTs: Triple Artemisinin-based Combination Therapies
TFPs: Technical and Financial Partners
UEMOA: West African Monetary and Economic Union /Union Economique et Monetaire Ouest Africaine
WHO: World Health Organization
